# Low-temperature and atmospheric pressure plasma for palm biodiesel hydrogenation

**DOI:** 10.1038/s41598-021-92714-x

**Published:** 2021-07-09

**Authors:** Grittima Kongprawes, Doonyapong Wongsawaeng, Kanokwan Ngaosuwan, Worapon Kiatkittipong, Suttichai Assabumrungrat

**Affiliations:** 1 Research Unit on Plasma Technology for High-Performance Materials Development, Department of Nuclear Engineering, Faculty of Engineering, Chulalongkorn University, 254 Phayathai Road, Pathumwan, Bangkok, 10330 Thailand; 2 Division of Chemical Engineering, Faculty of Engineering, Rajamangala University of Technology Krungthep, Bangkok, 10120 Thailand; 3 Department of Chemical Engineering, Faculty of Engineering and Industrial Technology, Silpakorn University, Nakhon Pathom, 73000 Thailand; 4 Center of Excellence in Catalysis and Catalytic Reaction Engineering, Department of Chemical Engineering, Faculty of Engineering, Chulalongkorn University, Bangkok, 10330 Thailand; 5 Bio-Circular-Green-Economy Technology and Engineering Center, BCGeTEC, Department of Chemical Engineering, Faculty of Engineering, Chulalongkorn University, Bangkok, 10330 Thailand

**Keywords:** Biodiesel, Chemical engineering

## Abstract

Partially hydrogenated fatty acid methyl ester (H-FAME) is conventionally produced through partial hydrogenation under high pressure and elevated temperature in the presence of a catalyst. Herein, a novel green, catalyst-free, non-thermal and atmospheric pressure dielectric barrier discharge (DBD) plasma was employed instead of a conventional method to hydrogenate palm FAME. H-FAME became more saturated with the conversion of C18:2 and C18:3 of 47.4 and 100%, respectively, at 100 W input power, 1 mm gas-filled gap size and 80% H_2_ in the mixed gas at room temperature for 5 h, causing a reduction of the iodine value from 50.2 to 43.5. Oxidation stability increased from 12.8 to 20 h while a cloud point changed from 13.5 to 16 °C. Interestingly, DBD plasma hydrogenation resulted in no *trans*-fatty acid formation which provided a positive effect on the cloud point. This green DBD plasma system showed a superior performance to a conventional catalytic reaction. It is an alternative method that is safe from explosion due to the mild operating condition, as well as being highly environmentally friendly by reducing waste and energy utilization from the regeneration process required for a catalytic process. This novel green plasma hydrogenation technique could also be applied to other liquid-based processes.

## Introduction

The increase in population, as well as advances in several technological areas, result in higher energy demands. One of the important sources is fossil fuel that has been used in transportation, electric power generation, agricultural machines, shipping, etc. Nowadays, the utilized fuel is fossil-based diesel or petrodiesel that cannot be replenished and has many negative effects such as highly toxic pollutants and greenhouse gases^1^. To reduce the use of petrodiesel, alternative fuels such as renewable energy: wind, solar and biofuel have been developed^2^.

Biodiesel is one of the renewable energy types that can be used in place of petroleum-based diesel. Typically, it can be synthesized from crop oil, animal fats or even waste cooking oil via several methods such as microemulsion, pyrolysis, esterification and transesterification^3^. On the commercial scale, transesterification is often utilized by mixing triglycerides in oils with short-chain alcohol catalyzed by appropriate catalysts which can be acid, base or enzyme in the homogenous or heterogeneous form at mild conditions^4,5^. Besides, the reaction can proceed in the absence of a catalyst, but a supercritical condition is required^6^. The final products are fatty acid methyl esters (FAMEs) and glycerol. Biodiesel can be mixed with petrodiesel in any proportion such as B5, B10 and B20, although neat biodiesel (B100) can be directly used in certain heavy diesel engines^7^. Biodiesel consists of very low carbon residue, no sulfur and about 10% oxygen which assists complete combustion. These advantages result in a significant reduction of emission and pollution: NO_x_, SO_x_ and dust particles, which drastically affect both climate change and human health^2^. However, biodiesel composes of long-chain carbon atoms containing C=C bonds, resulting in low oxidation stability compared to petrodiesel^8^.

The oxidation in biodiesel (FAME) can be initiated by light, heat or certain metals to generate free radicals. During the propagation process, the reaction between the free radical and oxygen in the air forms peroxide as an oxidation product. Additionally, the peroxide radical can react with a stable FAME to produce hydroperoxide which can continually generate hydroxyl radicals, water, as well as soluble and insoluble polymers. These oxidation products alter fuel properties: acid value, viscosity, cloud point and so on leading to degradation of engine parts^9,10^. There are multiple ways to delay the oxidation, for example, avoiding conditions prone to oxidation, storing in a suitable container, adding proper additives and using feedstocks with a low unsaturated composition^11,12^. For the last method, besides selecting an appropriate feedstock for biodiesel synthesis, a hydrogenation reaction can be performed on FAME to partially saturate the carbon chains.

Partial hydrogenation of fatty acid methyl ester (H-FAME) has been utilized to improve the ability to withstand oxidation. This method is to produce the more saturated FAME, specifically, to decrease the polyunsaturated compositions (C18:2, C18:3), for this kind of FAME composes of bis-allylic and allylic methylene groups which are readily reactive with both O* and H* free radicals^13^. In general, the hydrogenation process requires high temperature, high pressure and a suitable catalyst, which normally is a Group VIII metal: Ni, Co, Pd, Pt and Rh with or without proper support materials^14^. The proposed mechanism is that this reaction forms atomic hydrogen and breaks down the double bond, and then the H atoms react at the bond to form a single bond^14,15^. Hydrogenation not only greatly improves the oxidation stability of FAME, but also enhances other properties such as higher flash point, lower sulfur content and lower acid value^16^. However, one aspect that should be considered when biodiesel becomes more saturated is the degradation in the cold flow property: the cloud point and the pour point^8^. This property affects the phase of the fuel to become gum or gel at a higher temperature, which limits the use in cold-weather countries.

For catalytic hydrogenation of FAME, a Pd catalyst is widely used as it provides a high catalytic activity compared to other metals. As presented by Thunyaratchatanon et al.^17^, Pd/SiO_2_ was investigated at 120 °C and 0.4 MPa for 4 h. It was found that the oxidation stability was enhanced from 1.4 to 30.5 h and that the cloud point changed from 1 to 6 °C within the reaction time of 2 h. Besides, Thunyaratchatanon et al.^18^ improved the catalyst performance by adding a Mg modifier to form Pd-Mg/SiO_2_. This assisted the reaction to take place at a lower temperature of 80 °C at 0.4 MPa for 4 h. The results showed that the oxidation stability rose from 2 to 11 h and that the cloud point changed from 7 to 10 °C. Moreover, alkaline and alkaline earth metals such as Na, Ca or Ba can be used as modifiers to improve the conversion of polyunsaturated fatty acid^19^. In the case of H-FAME produced from palm FAME, a Pd catalyst on SBA-15 support was also practiced as reported in the study of Chen et al.^20^. The reaction conditions were 100 °C and 0.3 MPa for 2 h. The findings revealed that the oxidation stability was increased from 19.4 to 27.9 h and that the cloud point rose from 12 to 13 °C. The study of Ramayeni et al.^21^ was on using a Ni/C catalyst at 120 °C, 0.6 MPa and 2.5 h. The results revealed that Ni offered desirable results with the increase in oxidation stability of palm FAME from about 4.75 to 10.03 h while the cloud point was not reported.

According to the previous studies, the catalytic hydrogenation reaction must be performed at high pressure (0.3–0.6 MPa) and elevated temperature (80–120 °C). It perhaps faces problems with high operating and maintenance costs from the high hydrogen pressure environment, as well as a slow deactivation of catalyst performance from the deposition of carbon or metals on the catalyst surface^22^. To avoid these issues, one of the alternative methods is to utilize plasma technology. Plasma is the fourth state of matter consisting of active species including energetic electrons, positive ions, excited and neutral atoms/molecules. It can be generated by appropriately supplying sufficient energy to gas to create partially/fully ionized plasma^23^. Different types of plasma can be generated under various conditions such as at different pressures. When emphasizing atmospheric-pressure plasma, a dielectric barrier discharge (DBD) plasma is one of the many types of plasma that is easily generated and utilized. It can be produced by supplying the power from an AC or a pulsed high voltage with a frequency of 500 Hz to 500 kHz to two electrodes separated by a dielectric material which assists in preventing sparks and arcs causing local high temperature^24,25^. The reactive species occur in the electrode gap of typically 0.1–10 mm^26^. The DBD plasma discharges can be divided into filamentary and diffuse structures, but in most cases, it is filamentary or microdischarge. It is a small light stream between the electrodes and then expands radially when hitting the dielectric, forming a “foot” structure. The parameters affecting the characteristics and number of microdischarge are applied voltage, discharge gap size, specific capacitance of the barrier material, type of gas and pressure. The filamentary DBD is crucial because there are intense free radicals and reactive species generated in the plasma stream. Furthermore, it produces local heat. Typically, some heat is released from the electrodes, indicating that heat generation can occur when using a DBD plasma^24^. The heat and light/photon (UV–Vis) produced in a DBD plasma influence the performance of a plasma-assisted reaction. Therefore, it is widely used in several chemical production processes. and in many industries such as ozone generation, CO_2_ laser, excimer lamps^23^, as well as to assist a catalyst for syngas, ammonia or methanol production^27–29^. It is also practiced for liquid-phase hydrogenation of heavy oil and oil without a catalyst.

In the experiments of Hao et al.^30^, a low-temperature DBD plasma was employed in the cracking process for the value-added of heavy oil. The plasma was generated from 3 gases: N_2_, CH_4_ and H_2_. The findings were that the hydrogen plasma can enhance the yield of trap oil to approximately 19%, which was higher than those of the non-plasma processes by about 8–33%. Plasma was utilized to break down the H–H bond of hydrogen gas and to drive the hydrogenation reaction to form a light product. This low-temperature plasma can be produced at reduced or atmospheric pressure. Although the high temperature was still necessary for the decomposition of heavy oil, the required reaction temperature could be decreased, for example, from 420 to 380 °C. High-Voltage Atmospheric Cold Plasma (HVACP) was also used in the study of Yepez and Keener^31^. Hydrogenation of soybean oil was studied at ambient conditions in the absence of a catalyst. The gas used to generate plasma was a mixture of 5% H_2_ and 95% N_2_. It was found that at the reaction time of 12 h, soybean oil became more saturated and that its properties were similar to margarine. The iodine value was decreased from 133 to 92. The composition of saturated and monounsaturated fatty acids increased by 12 and 4.6%, respectively, whereas polyunsaturated fatty acids decreased by 16.2%. More importantly, there was no detectable *trans* fatty acid formation.

Margarine production from palm oil using DBD plasma was likewise experimented with by Puprasit et al.^32^. The optimal conditions were 15% H_2_ in a mixed gas (He and H_2_) and room temperature for 8 h of reaction time. The DBD plasma reactor could be used to successfully produce margarine with a similar texture to that of commercial margarine. However, *trans* fat was created by a small amount of about 1.4%. In our previous work^33^, H-FAME derived from soybean oil was successfully synthesized in a DBD plasma reactor. The best results appeared when using 35 mL FAME, 25%H_2_ in the He and H_2_ gas mixture, and ambient temperature under atmospheric pressure. It was found that the produced H-FAME contained methyl oleate (C18:1) as the dominant composition at the reaction time of 5.5 h. The iodine value reduced from 128 to 67.4 while the oxidation stability was reasonably improved from 2.13 to 10 h, and the cloud point changed from − 1 to 11 °C.

From the above research findings, plasma reactors can effectively break down gas molecules into constituent atoms and successfully engendered the hydrogenation reaction. The present work aims to investigate H-FAME production in a DBD plasma reactor at low temperature and atmospheric pressure without using a catalyst. FAME derived from palm oil, which is the main vegetable feedstock for global biodiesel production of about 35%^34^, was studied. The utilized DBD plasma hydrogenation reactor was an improvement to the previous study^33^ to have more production capacity via FAME recirculation, as well as higher plasma discharge power. The effects of parameters: power input, gas-filled gap size, H_2_ concentration, reaction temperature and reaction time, were evaluated. This plasma treatment technique is highly environmentally friendly, for a catalyst is not required. This helps to reduce the processes of catalyst preparation and removal from the product. Also, since plasma hydrogenation occurs at low temperature and atmospheric pressure, the energy requirement, as well as the maintenance cost, can be reduced. This novel and green technology has a promising potential to be applied for hydrogenation of other high-value liquids, as well as for large-scale H-FAME production.

## Methodology

### Materials

Palm FAME was obtained from an industrial plant in Thailand. The gases used for H-FAME production, H_2_ and He, were of Ultra High Purity (UHP) grade procured from Alternative Chemical Company Ltd., Thailand. FAME yield and composition were analyzed by Gas Chromatography (GC) using methyl heptadecanoate and n-heptane of analytical reagent grade purchased from Sigma-Aldrich. The following chemicals were used for the determination of iodine value, peroxide value and acid number: cyclohexane (99.5%) of analytical reagent grade purchased from Loba Chemie Pvt., Ltd., sodium thiosulfate 5-hydrate (99.5–101%) obtained from KEMAUS, potassium iodide (99%) obtained from Ajax Finechem, a starch solution made from corn starch of Super-Fine brand purchased from a local department store, Wijs solution prepared from iodine mono-chloride (98%) and glacial acetic acid (99.7%) purchased from Panreac Applichem and QReC, respectively, chloroform (99.8%) and phenolphthalein indicator obtained from RCI Labscan, ethyl alcohol (95%) purchased from Samchai Chemical, Thailand, and sodium hydroxide (97%) of analytical reagent grade obtained from Loba Chemie Pvt., Ltd. All chemicals were used as received.

### Experimental set-up

**Figure 1:**
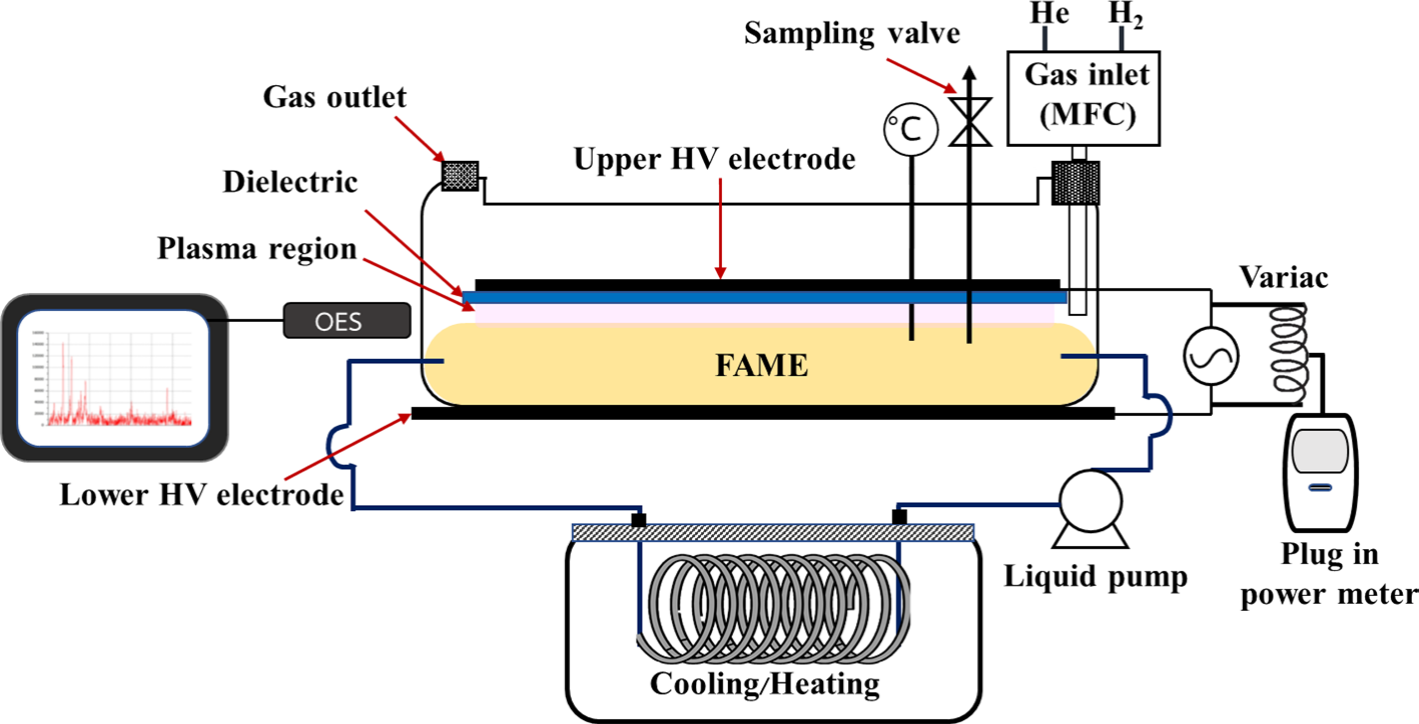
Drawing of constructed DBD plasma reactor (drawn using Microsoft PowerPoint version 2104, https://www.microsoft.com/th-th/microsoft-365/powerpoint).

Figure 1 presents a drawing of the DBD plasma hydrogenation system used in the present research. The reaction chamber was made of borosilicate glass with a volume of 4 L and a thickness of 5 mm. The DBD plasma system comprised two electrodes made of aluminum plates with the upper one (20 × 16 cm) placed inside the reaction chamber and the lower one (24.5 × 17.5 cm) sealed to the bottom of the chamber using high-temperature silicone. A glass dielectric sheet (22 × 17 cm) was attached to the bottom of the upper electrode using a high-temperature silicone sealant to prevent any aluminum contamination in FAME. The two electrodes were electrically connected to a high-voltage, high-frequency neon sign transformer of NeonPro brand, model MXP-15000–40, which provided a maximum output of 15 kV and 40 mA at 20 kHz. Although the transformer was a commercial neon sign transformer, with its suitable electrical output characteristics, it was applied to effectively generate DBD plasma with a maximum power of 100 W. The transformer used in the study of Kongprawes et al.^33^ had a maximum output of 10 kV, 30 mA, 25 kHz, thus, the present research presented an improvement to the previous work. Although the utilized transformer had no output adjustment knob, it was found that by connecting a variac directly to the transformer input, the output power can be easily regulated by adjusting the variac setting. The total power consumption of the neon sign transformer was measured by a plug-in power meter connected to the variac. The accumulated power consumption was recorded and presented in the unit of kW-h. The waveform of the discharge voltage was measured by a high voltage probe connected to a digital oscilloscope (Tektronix TDS 2012). The gas mixture of H_2_ and He was controlled by Unit Instruments mass flow controllers models UFC-1000 for H_2_ and UFC-1260A for He. The mixed gas was supplied into the reaction chamber with the opening slightly below the glass dielectric plate (but not submerging into the FAME layer) to allow effective gas displacement in the gas-filled gap as shown in supplementary materials Fig. S1. To allow a large FAME treatment volume while maintaining a thin FAME layer inside the chamber, as well as to allow sufficient mass circulation in the chamber, FAME was pumped into and out of the chamber via a peristaltic pump. The FAME line external to the chamber was connected to a stainless steel coil placed inside a water bath to allow reaction temperature control. A thermometer and a sampling port were installed to observe the temperature and to perform online sample collection. Reactive species produced in the plasma were observed using a spectrometer (Ocean Optics, USB4000 model, wavelength range approximately 200–1050 nm, XR1-500 line grating blazed at 250 nm and L4 lens) connected to a fiber optic cable to collect the plasma photons. The spectrometer was connected to a PC-based OceanView software version 1.6.7 (https://www.oceaninsight.com/support/software-downloads/oceanview-software-downloads/) for visualization.

### Hydrogenation of FAME

For each batch, 300 mL of FAME was plasma hydrogenated. The plasma was generated from a H_2_/He gas mixture with an overall flow rate of 1 L/min. FAME was continuously circulated with a constant flow rate of 400 mL/min. He gas was selected as the inert gas for this study because plasma could be easily generated all over the electrode with few microfilament formations as explained in supplementary materials Sect. 1. The experimental investigation is shown in Fig. S2. The system was initially purged with a He gas with a flow rate of 3 L/min for 2 min to completely remove air present in the reaction chamber. Before plasma generation, the correct H_2_: He ratio was achieved by gradually increasing the H_2_ flow rate while slowly reducing the He flow rate. When the gas was at the desired proportion, the neon power supply was energized to generate plasma. The studied parameters were the consumed power of the DBD power supply (50–100 W), gas-filled gap size (1–5 mm, which was the small clearance between the lower surface of the glass sheet and the surface of the FAME inside the chamber), concentration of H_2_ (25–80 vol.%), FAME temperature (20–60 °C) and reaction time. The thickness of the FAME layer inside the chamber was identical for every run, and the gas-filled gap size was manually adjusted by configuring the length of the small polyethylene supporting columns at the four corners of the glass plate. For the investigation on optimal parameters, the reaction took place for 1 h without water in the water bath except for the temperature study case. After the reaction, H-FAME was stored in a plastic container at ambient temperature and covered with nitrogen gas to avoid oxidation. Plasma hydrogenation experiments were performed in duplicate and the reported values represented the average with the error bars showing the standard deviation.

### FAME and H-FAME analysis

FAME yield and fatty acid compositions were analyzed according to EN14103 standard^35^ using gas chromatography (Shimadzu GC-2010 Plus with a DB-WAX capillary column equipped with a flame ionization detector using helium carrier gas). The introduced volume of the sample was 1 μL. The detector temperature was 250 °C with a split ratio of 1/50. The temperature was increased from 150 to 220 °C at the rate of 3 °C/min with a holding time of 5 min. FAME yield and composition were calculated from Eqs. (1) and (2), respectively.

1 $${\text{FAME~}}\;{\text{yield~}}(\% ) = \frac{{\Sigma {\text{A}} - {\text{A}}_{{{\text{IS}}}} }}{{{\text{A}}_{{{\text{IS}}}} }} \times \frac{{{\text{C}}_{{{\text{IS}}}} \times {\text{V}}_{{{\text{IS}}}} }}{{\text{m}}} \times 100\%$$

where ΣA = total peak area, A_IS_ = internal standard (methyl heptadecanoate) peak area, C_IS_ = concentration of internal standard solution (mg/mL), V_IS_ = volume of internal standard solution (mL) and m = mass of sample (mg).

2 $${\text{X}}_{{{\text{composition}}}} (\% ) = \frac{{{\text{A}}_{{\text{X}}} }}{{\Sigma {\text{A~}} - {\text{~A}}_{{{\text{IS}}}} }} \times 100\%$$

where ΣA = total peak area, A_x_ = type of FAME composition peak area, e.g., methyl palmitate (C16:0), methyl stearate (C18:0) and methyl oleate (C18:1), A_IS_ = internal standard (methyl heptadecanoate) peak area.

Since the DB-WAX capillary column cannot detect the *trans*-configuration, to determine the *trans* quantity and functional groups of the feed compared to the final product, Perkin Elmer Spectrum One Fourier Transform Infrared (FTIR) was applied. The FTIR conditions were: Universal Attenuated Total Reflectance (UATR) sensor technique, resolution of 4.0 cm^−1^, scan range of 4000–515 cm^−1^ and number of sample scans of 64. Feed FAME and H-FAME obtained from the optimal condition were analyzed for the compositions from C8 to C20 by Gas Chromatograph- Mass Spectrometer (GC–MS) of 78,908 GC -5977A MSD, Agilent, USA. The test technique was Gas Chromatography-Electron Ionization/ Mass Spectrometry (GC-EI/MS). The level of saturation indicated by iodine value was investigated by the Wijs-cyclohexane method, ASTM D1959^36^. Acid number and peroxide value were measured according to the standards of AOAC, 1997 and AOCS, 1997, respectively. Oxidation stability of FAME and H-FAME were analyzed by EC Meter, EC-450L, Istek Inc., Korea, following WI-RES-EC Meter-001 and the in-house method based on the modified Rancimat test (EN 15,751:2009)^37^. In addition, Walter Herzog GmbH, Germany was utilized to determine the cloud point as in the standard of ASTM-D-2500^38^.

## Results and discussion

### FAME properties

As-received palm FAME showed a yield of 97.9%. It consisted of saturated and monounsaturated FAMEs as the main composition: 41.2 ± 0.6% methyl palmitate (C16:0), 3.6 ± 0.2% methyl stearate (C18:0) and 41.8 ± 0.1% methyl oleate (18:1). The polyunsaturated FAMEs were composed of methyl linoleate (C18:2) and methyl linolenate (C18:3) of about 9.7 ± 0.3 and 0.2 ± 0.1%, respectively. This composition resulted in the oxidation stability of 12.8 h without adding any additive, while its cloud point was 13.5 °C. The iodine value and acid number were 0.5% g I_2_/g and 0.2 mg KOH/g, respectively.

### Effect of input power

**Figure 2:**
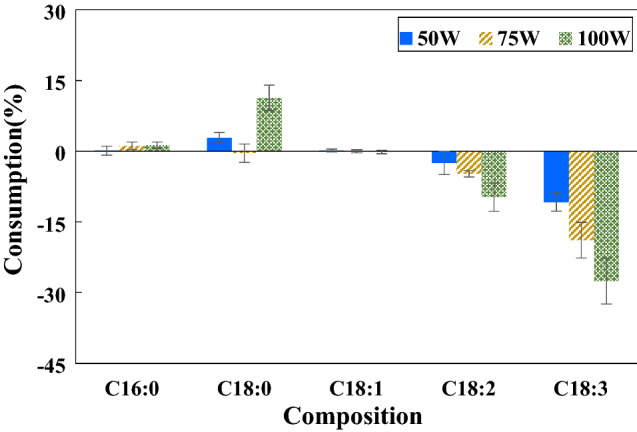
Effect of input power on FAME composition changes (1 mm gap and 25%H_2_ at room temperature for 1 h).

The studied input power was 50, 75 and 100 W. The gas-filled gap of 1 mm was configured. The reaction was conducted using 25%H_2_ in the gas mixture at room temperature (starting at 25 °C and rising to about 38 ± 2 °C due to the heat from the DBD plasma^39^). The sine waveform of the discharge voltage is displayed in supplementary materials Fig. S3. It indicated that more input power resulted in higher discharge voltage, while the frequency exhibited a minute fluctuation between 19.01 and 19.40 kHz. Supplying 50, 75 and 100 W of input power resulted in the peak-to-peak discharge voltage of 1.76, 1.84 and 2.1 kV, respectively. These closed-circuit voltages were much lower than the 15 kV open-circuit voltage rating of the utilized neon transformer, which was according to the expectation. Figure 2 presents the composition changes at the reaction time of 1 h. The consumption percentage referred to the conversion of the C=C bond into a single bond compared to the feed. The results revealed that applying 100 W of input power provided the highest conversion of polyunsaturated FAME followed by 75 and 50 W, respectively. The reduction of C18:2 and C18:3 from using 100 W was 9.3 (from 9.7 ± 0.3 to 8.8 ± 0.2%) and 27.6% (from 0.2 ± 0.1 to 0.1 ± 0.0%), respectively. In addition, saturated FAME increased as follows: 1.4% for C16:0 (from 41.2 ± 0.6 to 41.8 ± 0.3%) and 11.4% for C18:0 (from 3.6 ± 0.2 to 4.0 ± 0.1%). The response of FAME with input power was according to the expectation–high input power provided higher energy to produce more plasma density as well as more intense filamentary discharges with a high concentration of electrons^40^. The generated energetic electrons could transfer the energy to activate the C=C bonds of FAME as well as to other gas atoms/molecules generating more reactive species. However, microfilaments caused local heating. In the present work, the temperature of FAME near the microfilaments was measured to be about 5 °C higher than that in other areas. In conclusion, high input power resulted in high plasma density generating a large amount of atomic hydrogen to readily react with the C=C bonds.

### Effect of gas-filled gap size

**Figure 3:**
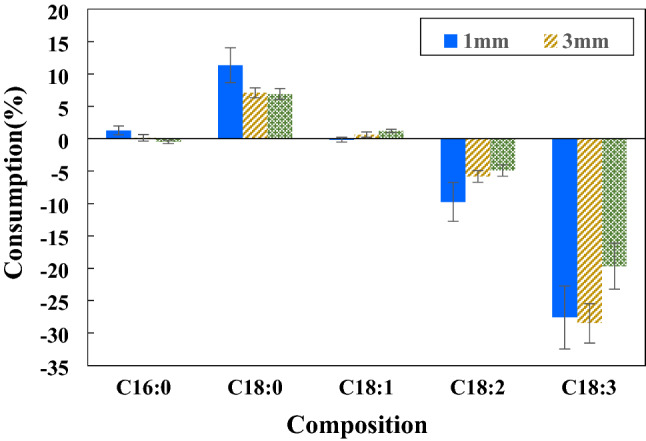
Effect of gas-filled gap on FAME composition changes (100 W and 25%H_2_ at room temperature for 1 h).

The gap size was configured at 1, 3 and 5 mm. The conditions were 100 W, 25% H_2_ and room temperature. The reaction took place for 1 h. The discharge voltage for each gap size was measured for the power of 50–100 W. The different gap sizes resulted in dissimilar voltages under the same input power as presented in supplementary materials Fig. S4. Using the larger gap size, the discharge voltage became higher. For the case of 100 W, the peak-to-peak voltage at 1, 3 and 5 mm gap size was 2.10, 2.46 and 2.96 kV, respectively. This behavior was expected as a larger gap size resulted in the system becoming more open circuit electrically. For a very large electrode gap, the peak-to-peak voltage would become the neon transformer’s open-circuit voltage rating of 15 kV. When considering the percent consumption of the C=C bonds including C18:2 and C18:3, it was found that the smallest gap of 1 mm offered the best overall result with a significant decrease in C18:2 and C18:3 followed by 3 and 5 mm, respectively as presented in Fig. 3. The reduction of C18:2 and C18:3 obtained from the 3 mm gap was 6.2 (from 9.7 ± 0.3 to 9.1 ± 0.1%) and 28.5% (from 0.2 ± 0.1 to 0.1 ± 0.0%), respectively. For 5 mm, C18:2 and C18:3 decreased by 5.2 (from 9.7 ± 0.3 to 9.2 ± 0.1%) and 19.7% (from 0.2 ± 0.1 to 0.1 ± 0.0%), respectively. The gas-filled gap influenced the performance of the plasma catalyzed reaction. Being characteristic of the DBD plasma, the smaller the gap between the two electrodes, the denser the generated microfilament discharges and the resulting higher plasma intensity, and vice versa. On the contrary, a smaller gap might impede gas flow and might result in a diminished quantity of atomic hydrogen. For the plasma to be generated, the larger gas gap required a larger amount of supplied energy to exceed the breakdown voltage (V_b_) of the gas within the gap. The breakdown voltage of a gas is the function of pressure (p) and gap distance (d). According to Paschen’s curve for the He-H_2_ gas mixture presented in the study of Das et al.^41^, the value of pressure times gap distance of the present work should be 76, 228 and 380 Torr·cm for the gap size of 1, 3 and 5 mm, respectively. According to Paschen’s curve in supplementary materials Fig. S5, it showed that the bigger gap necessitated the higher breakdown voltage of gases. This implied that the applied voltage for the case of a small gap exceeded the voltage required to dissociate and ionize the gas molecules. Furthermore, a small gap resulted in a short distance for atomic hydrogen to travel and react with FAME, thus, more hydrogen atoms/ions could react with FAME before recombination to become hydrogen molecules ineffective for hydrogenation. With the result showing the smallest gap of 1 mm performing the best, the effect of higher plasma intensity and short travel distance for hydrogen radicals must have outweighed the effect of gas flow impediment if any.

### Effect of H_2_ concentration

Firstly, the H_2_ percentage was increased until plasma could not be sustained. The highest value was slightly over 80% when the plasma visually ceased to exist, confirmed by a sudden drop in the transformer power input (no plasma generation meant no power drawn by the transformer, a phenomenon similar to an AC transformer with an open circuit on the secondary winding which would draw no current on the primary side). The clearly audible high-frequency sound characteristic of a DBD plasma also went silent. Thus, the H_2_ concentration was examined at 25, 52.5 and 80%. The most appropriate gas-filled gap of 1 mm and the input power of 100 W at room temperature were used. As the solubility of H_2_ in biodiesel was very low, H_2_ uptake by the biodiesel in the reaction chamber was negligible. As presented in the study of Tomoya et al.^42^, H_2_ can be fairly dissolved in bio-oil. For example, H_2_ was dissolved in triolein (triglycerides with one unit of glycerol and three units of oleic acid) at a mole fraction of 0.1323 at about 80 °C and 7.5 MPa. It was also reported that H_2_ solubility increased with pressure. This demonstrates that there was a very small amount of H_2_ incorporated into the liquid phase in this low-pressure and low-temperature treatment regime. Thus, the reaction was two-phase (gas/liquid) that occurred at the plasma-FAME interface. Since the interfacial area remained unaffected with different H_2_ concentrations, any observed effect on FAME composition changes reflected the effect of H_2_ concentration.

**Figure 4:**
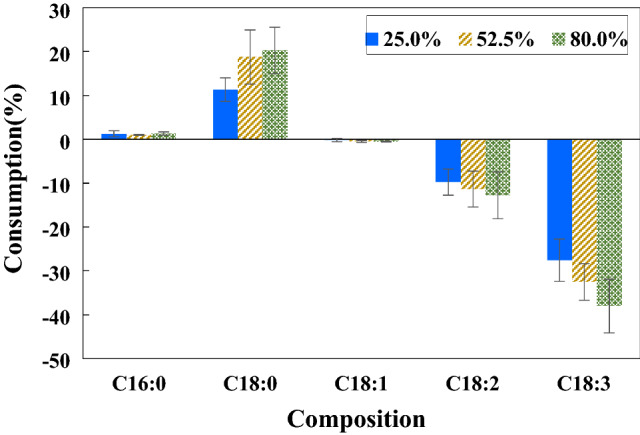
Effect of H_2_ concentration on FAME composition changes (100 W and 1 mm gap at room temperature for 1 h).

As shown in Fig. 4, the highest H_2_ concentration of 80% appeared to show the highest conversion of polyunsaturated FAMEs, followed by 52.5 and 25%, respectively. For the case of 80%H_2_, C18:2 and C18:3 were decreased by about 13.4 (from 9.7 ± 0.3 to 8.4 ± 0.4%) and 38.0% (from 0.2 ± 0.1 to 0.1 ± 0.0%), respectively, whereas saturated FAME increased as follows: C16:0 by 1.4% (from 41.2 ± 0.1 to 41.8 ± 0.2%) and C18:0 by 20.4% (from 3.6 ± 0.2 to 4.4 ± 0.2%).

### Effect of reaction temperature

**Figure 5:**
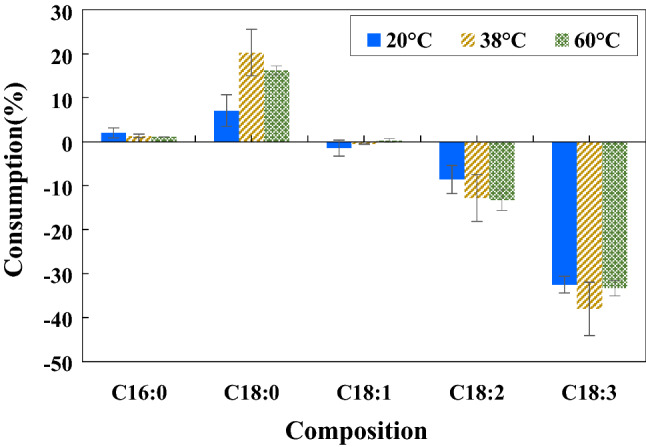
Effect of reaction temperature on FAME composition changes (100 W, 1 mm gap and 80%H_2_ for 1 h).

The temperatures of 20 ± 2 °C, 38 ± 2 °C (due to plasma heating only), and 60 ± 2 °C were investigated. The reaction conditions were 100 W input power, 1 mm gas-filled gap and 80%H_2_. Figure 5 demonstrates the effect of temperature on H-FAME composition, which revealed that temperature played no significant role. High temperature could not enhance the reaction speed, while low temperature could not amplify the benefit of the exothermic hydrogenation. The gas outlet temperature was also measured to be about 22.5 ± 0.7 °C (initially at 18 °C and became constant after plasma application for 15 min, for the case of the FAME temperature of 38 ± 2 °C). This implied that reduced or elevated temperature was not required for plasma hydrogenation as also revealed in the previous work^33^. The energy required to ionize and split hydrogen molecules and to activate the double bonds of FAME relied on the applied voltage supplied from the neon sign transformer and not on thermal energy (for the studied DBD plasma system), unlike conventional chemical catalysis that the reaction normally required sufficient thermal energy to overcome the activation energy. Although the plasma channels where the plasma chemical reactions take place are usually at elevated temperatures^43^, the elevated temperature was beyond the scope of the present study because performing the plasma treatment at ambient temperature is highly energy-efficient as well as cost-effective since no heating or cooling system is required.

### Effect of reaction time

**Figure 6:**
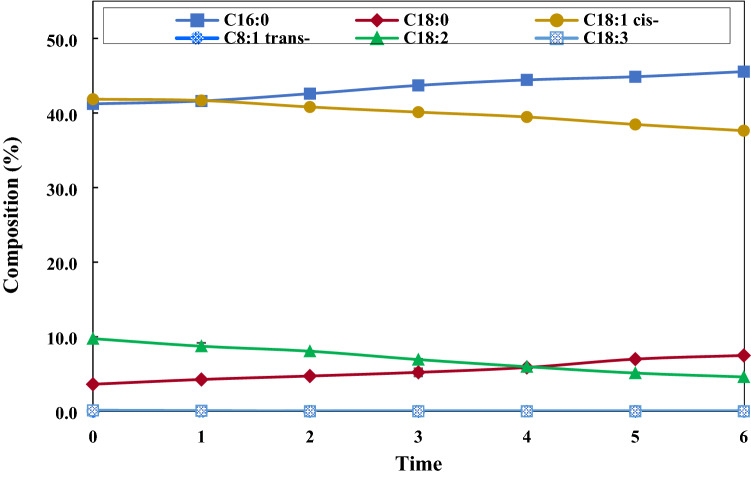
Effect of reaction time on FAME composition changes (100 W, 1 mm gap and 80%H_2_ at room temperature).

Catalyst-free hydrogenation was performed up to 6 h under the optimal parameters (100 W 1 mm gap and 80%H_2_ at room temperature), with Fig. 6 displaying the changes of FAME composition. The unsaturated FAMEs were hydrogenated resulting in the saturated ones accumulating over time. H-FAME at 6 h of reaction time composed of 45.5 ± 0.1% C16:0, 7.5 ± 0.1% C18:0, 37.6 ± 0.1% C18:1 and 4.6 ± 0.1% C18:2, while C18:3 completely reacted with hydrogen atoms. When considering the bond dissociation energy (BDE) in a normal alkane, the **CH**_**3**_–nC_i_H_2i+1_ bonds are the strongest with BDE of about 364.0 kJ/mol, while the **C**_**2**_**H**_**5**_–nC_i_H_2i+1_ bonds are the weakest of about 359.8 kJ/mol, with the bold letters referring to the dissociated atoms^44^. The ethyl group (–C_2_H_5_) in C18:0 could possibly cleavage, causing an increase in C16:0.

The oxidation stability increased with increasing hydrogenation duration due to FAME becoming more saturated. However, FAME at 6 h of plasma treatment was slightly over hydrogenated for its cloud point was 16.5 °C, which was marginally above the biodiesel standard of Thailand (16 °C maximum). It was found that the optimal hydrogenation time to satisfy the Thai cloud point standard was 5 h and that the compositions of the final product measured by GC–MS contained saturated FAMEs as the largest component of about 54.1%. Besides, there were mono- and polyunsaturated FAMEs of 38.8 and 5.1%, respectively. Feed FAME and H-FAME at 5 h of reaction time were analyzed by GC–MS. It was found that both consisted of carbon chains of fatty acid of methyl esters from C8 to C20. The main composition changes were C16 and C18. Table 1 shows the detected compositions with the bolds representing the total saturated, monounsaturated and polyunsaturated compositions in each FAME. When considering the results obtained in catalytic hydrogenation of palm-based FAME using Pd/SBA-15 in the study of Chen et al.^20^, the reported FAME composition was saturated FAME, mono-, di- and tri unsaturated FAMEs as presented in Table 1. The conversion of di-unsaturated FAME and tri-unsaturated FAME was 37.1 and 63.2%, respectively, after 2 h of reaction time. To achieve a similar level of conversion, the DBD plasma system needed to be conducted for 4 h. At this time, the conversion of C18:2 and C18:3 was 38.7 and 100%, respectively. However, a direct comparison of reaction efficacy between a physical catalyst and plasma catalysis cannot be readily made because of different FAME volumes used, as well as different types and amounts of energy supplied into each system. Chen et al.^20^ studied a continuous process of 0.37 g/min, so for 2 h, the treated volume was 53.5 mL. The total volume used in the present study was 300 mL, which was about 5.6 times higher. The DBD plasma could also take place at ambient conditions and did not require a catalyst, which eliminated the problems of catalyst deactivation and material degradation due to high pressure and high-temperature operation.

**Table 1:** Compositions of FAME and H-FAME determined by GC–MS (100 W, 1 mm gap, 80%H_2_, room temperature and 5 h) compared to catalytic hydrogenation.

Composition (%)	Present work		Ref. Chen et al.^20^	
	FAME	H-FAME	FAME	H-FAME
Saturated FAMEs	**46.72**	**54.08**	**49.07**	**51.35**
Methyl caprylate C8:0	0.01	0.03	–	–
Methyl caprate C10:0	0.02	0.01	–	–
Methyl laurate C12:0	0.22	0.28	–	–
Methyl myristate C14:0	1.08	1.40	–	–
Methyl pentadecanoate C15:0	0.04	0.03	–	–
Methyl palmitate C16:0	41.22	44.86	–	–
Methyl margarate C17:0	0.10	0.08	–	–
Methyl stearate C18:0	3.62	6.99	–	–
Methyl eicosanoate C20:0	0.41	0.40	–	–
Monounsaturated FAMEs	**42.30**	**38.83**	**41.01**	**42.27**
Total *cis-*	42.30	38.83	40.90	34.47
Total *trans-*	**–**	**–**	0.11	7.73
Methyl palmitoleate C16:1 (*Cis*-)	0.23	0.13	–	–
Methyl oleate C18:1 *(Cis-)*	41.85	38.46	–	–
Methyl oleate C18:1 (*Trans-)*	–	–	–	–
Methyl eicosenoate C20:1 *(Cis-)*	0.22	0.24	–	–
Polyunsaturated FAMEs	**9.93**	**5.12**	**9.73**	**6.07**
Methyl linoleate C18:2/di-unsaturation	9.73	5.12	9.54	6.00
Methyl linolenate C18:3/tri-unsaturation	0.20	0.00	0.19	0.07

### Plasma hydrogenation of FAME mechanism and reactive species generated during reaction

When He and H_2_ gases received sufficient energy from the applied high voltage, electrons can be stripped from molecules/atoms. This caused the formation of active species including energetic electrons, neutral and excited molecules/atoms, as well as positive ions. The He gas was also excited and ionized, but it was still inert and did not react with FAME. However, the reactive species of He still played an important role in the reaction because many possible reaction channels took place from this gas such as momentum transfer, dimer-induced dissociative ionization, ion–electron recombination and so on^45^. This assisted to produce more He species and free electrons that could transfer the energy to H_2_ to maintain the plasma production. In addition, the FAME could be activated by these reactive species instigating methyl ester radical’s formation. Then, the ions and energetic electrons collided with other atoms/molecules in a stable/excited state to generate more hydrogen ions (H^+^)/radicals (H^•^) and other electrons which is called a Penning ionization. In a cold hydrogen plasma, it was reported that the generated reactive species were H^+^, H^3+^ and H^•^ of 0.0001%, 0.1%, and 1%, respectively. The system was rich in H^•^, so they should most participate in the hydrogenation of FAME^46^.

**Figure 7:**
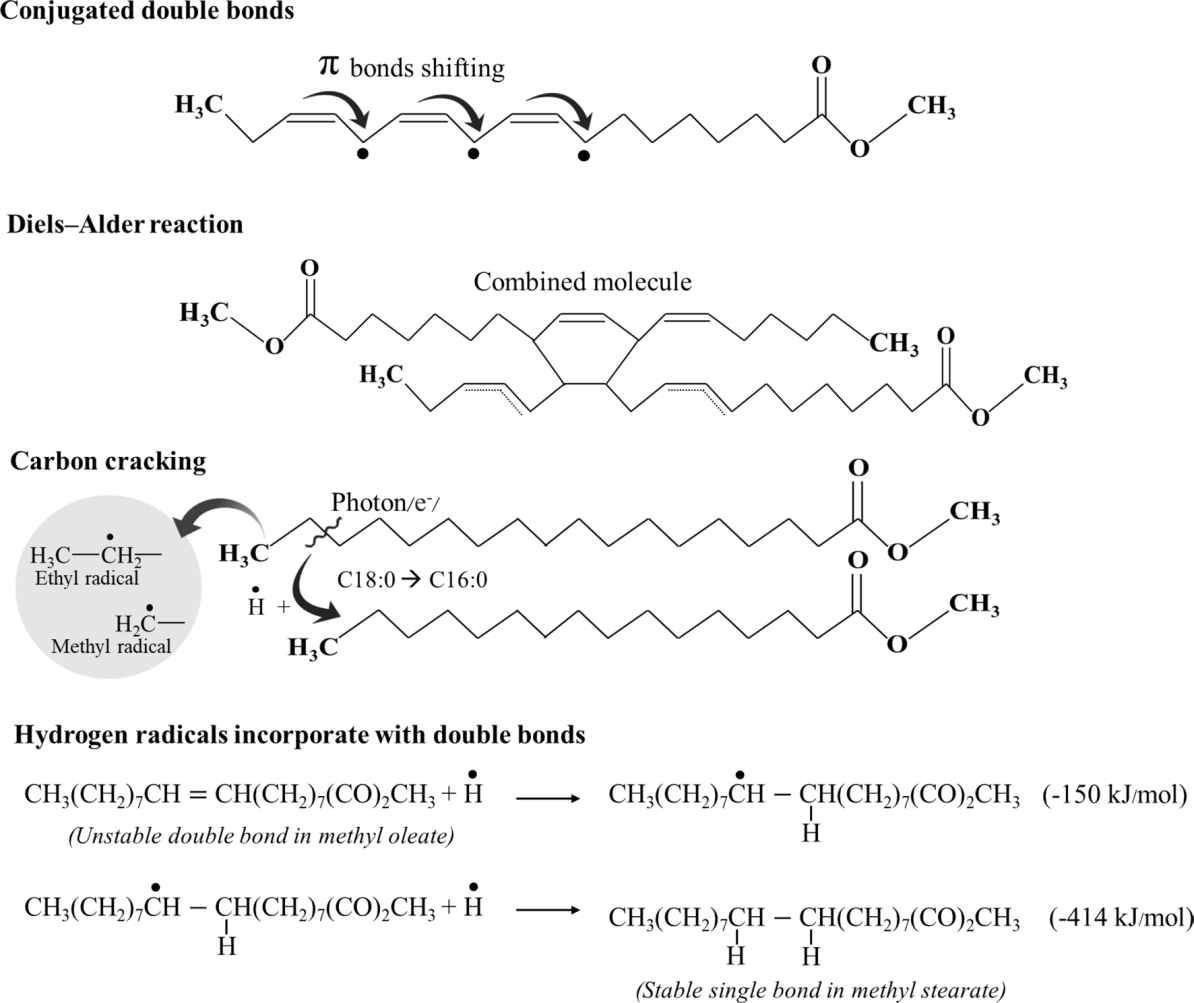
Plasma chemical mechanism for FAME hydrogenation (drawn using Microsoft PowerPoint version 2104, https://www.microsoft.com/th-th/microsoft-365/powerpoint).

The chemical affinity of the C=C bonds or alkene is unstable, for they readily react with a substance or allow the addition of hydrogen atoms to become stable. Polyunsaturated FAMEs (C18:2, C18:3) are conjugated double bonds. They could be easily activated by the plasma light or plasma active species. This causes the hydrogen atom to be dislodged, especially at bis-allylic and allylic positions which are extremely weak, and the methyl ester radical to be generated results in double bonds shifting. Since the bonds were moving, polymerization or Diels–Alder condensation between FAME molecules was possible to be initiated. The carbon cracking initiated by photon/energetic electrons was able to occur in the plasma process^31–33^, particularly at the C_2_H_5_–nC_i_H_2i+1_ bonds in alkene which are weakest as explained in Sect. 3.6. This produced ethyl and methyl radicals^22^ from eighteen carbon chains (C18) to form sixteen carbon chain (C16) radicals. After that, the C16 radicals accepted the hydrogen radicals to form stable fatty acid methyl esters. In this work, the scission of C18 resulted in an increase in C16:0 by about 3.6%. Although the C16 radicals could incorporate with the methyl and hydrogen radicals to become seventeen carbon chains, C17 was not detected from the GC–MS results. As for the hydrogenation of FAME, the hydrogen radicals were added to the π-double bonds at the plasma-FAME surface. For one hydrogen radical to establish a bond with carbon resulting in -CH radical formation, 150 kJ/mol of energy was required^47^. Afterward, another H^•^ was combined, with the required energy of 414 kJ/mol^48^, to create a stable single bond as depicted in Fig. 7. Most of the reaction taking place in the DBD plasma system was hydrogenation to transform mono- and unsaturated compositions into saturated ones which were C18:0 (from 3.6 to 7.0%) and C16:0 (from 41.2 to 44.9%).

**Figure 8:**
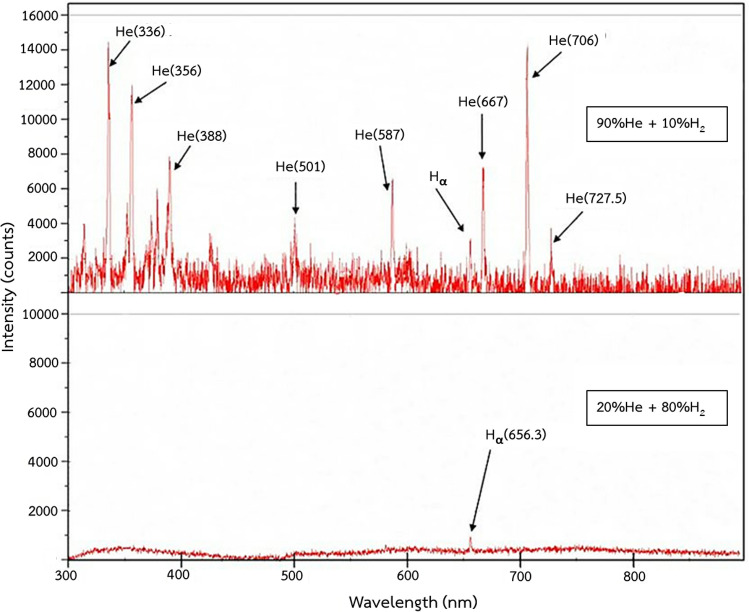
Optical emission spectra of He and H_2_ plasma (100 W and 1 mm gap at room temperature).

To observe the reactive species generated from the He-H_2_ gas mixture and to detect the reactive species from other elements especially from carbon and hydrocarbon as some eighteen-carbon chains could be transformed into sixteen-carbon chains during plasma processing, reactive species were monitored by optical emission spectroscopy (OES) as presented in Fig. 8 The reaction conditions were: input power of 100 W and gas-filled gap of 1 mm at room temperature. The difference in H_2_ percentage in the mixed carrier gas resulted in dissimilarly observed peaks. At 90%He and 10%H_2_, all peaks of He species were clearly detected at 336, 356, 388, 501, 587, 667, 706 and 727.5 nm while only one peak of H_2_ species appeared which was H_α_ at 656.3 nm representing hydrogen atom excitation. This was related to the characteristic of cold plasma presented in the study of El-Zeer et al.^49^ and mentioned in Yepez et al.^46^’s work that cold plasma consisted mostly of hydrogen radicals responding in the reaction. For the case of 20%He and 80%H_2_, the plasma color was visually observed to became brighter/lighter, having a more whitish tone as shown in supplementary materials Fig. S6. The acquired plasma spectrum showed only the presence of H_α_ species. These results verified that He and H_2_ reactive species were indeed generated in the system. Besides, there was no appearance in optical emission spectra of other strong peaks corresponding to a generation of other reactive species, carbon and hydrocarbon.

### FTIR analysis

**Figure 9:**
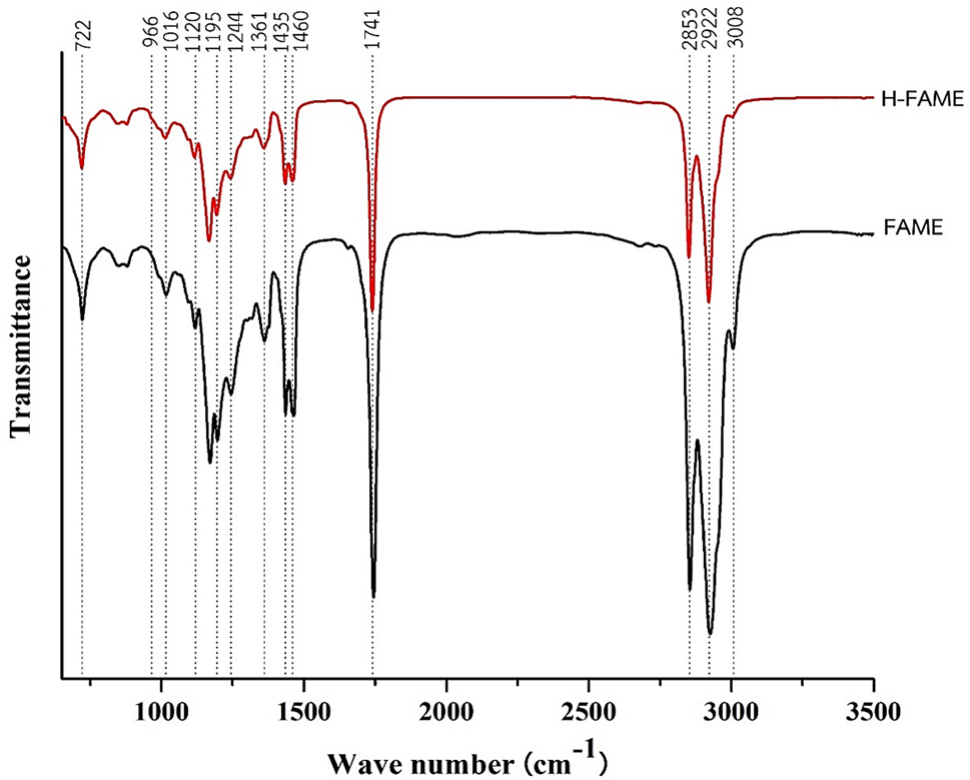
FTIR spectra of FAME and H-FAME (100 W, 1 mm gap and 80%H_2_ at room temperature).

The chemical functional groups were examined by FTIR as shown in Fig. 9. Both FAME and H-FAME consisted of a peak at 3008 cm^−1^ representing the unsaturated fatty acid methyl esters, C=CH stretching, and the peak decreased due to hydrogenation. The peaks at wave numbers 2922 and 2853 cm^−1^ were the asymmetric and symmetric stretching vibration of the alkane group, C–H, respectively. The strong peak at 1741 cm^−1^ corresponded to the ester group, C=O stretching. In addition, the asymmetric stretching of CH_3_ was detected at wavenumbers 1435 and 1460 cm^−1^ while CH_2_ was represented at 1361 cm^−1^. The peak at 1195 cm^−1^ indicated O–CH_3_ stretching which was methyl esters. Besides, C–O anti-symmetric and C–O symmetric vibrations were present at 1016 and 1169 cm^−1^, respectively. The peaks at 1244 and 1120 cm^−1^ corresponded to C–O and C–O–C stretching. The characteristic peaks of *cis*- and *trans*-configurations appeared at 722 (*cis*) and 966 (*trans*) cm^−1^. *Cis-*can be normally detected in FAME and H-FAME, while *trans-*should not be present in FAME, for it was synthesized from edible oil^50^. Most importantly, no peak at 911 cm^−1^ was found—no *trans* fatty acid methyl ester formation from the hydrogenation reaction using low-temperature DBD plasma.

### H-FAME properties

FAME and H-FAME properties compared to biodiesel standards were demonstrated in Table 2. Feed FAME has high oxidation stability of 12.8 h with the cloud point somehow exceeding the ASTM D6751 requirement. After 5 h of plasma treatment (100 W, 1 mm gap, 80%H_2_, room temperature), it achieved 20 h of oxidation resistance with an increase of the cloud point from 13.5 to 16 °C. If 16.5 °C of the cloud point was allowed following 6 h of hydrogenation, the oxidation stability would be higher than 20 h. The more saturation level caused the reduction of iodine value from 50.2 to 43.5, verifying that the DBD plasma system could be practiced for effective H-FAME production. It offered superior performances to a catalytic reaction as presented in the study of Chen et al.^20^. In the case of using 0.5 wt.% of the Pd/SBA-15 catalyst at 100 °C, 0.3 MPa for palm H-FAME production, the oxidation stability increased by 8.5 h (from 19.4 to 27.9 h) with a small change in the cloud point from 12 to 13 °C after 2 h of hydrogenation. A conversion of C18:2 and C18:3 was about 37.11 and 63.16%, respectively, while the saturated- and monounsaturated compositions rose by 4.65 and 3.07%, respectively. There was also *trans-* formation of about 7.73% (per 100% H-FAME content) after the reaction. The composition still consisted mostly of C18:1, and this was perhaps the reason why the cloud point increased by only 1 °C. For the results obtained in the present study, every FAME composition with the C=C bond was hydrogenated into a single bond to a varying degree. The conversion of C18:1, C18:2 and C18:3 was 8.1, 47.4 and 100%, respectively, increasing the saturated FAME by 16.6%. The product could resist oxidation by an additional 7.2 h. However, due to the higher amount of saturated FAME, the increase in the cloud point was greater than that of the catalytic reaction.

**Table 2:** Properties FAME and H-FAME compared to biodiesel standards (100 W, 1 mm gap, 80%H_2_, room temperature and 5 h).

Properties	FAME	H-FAME	Standard
Oxidation stability (h)	12.8	20.0	> 6.0^a^, 3.0^b^, 10.0^c^
Cloud point (°C)	13.5	16.0	< 12.0^b^,16.0^c^
Iodine value (gI_2_/100)	50.2	43.5	< 120.0^a,b,c^
Acid number (mgKOH/g	0.3	0.3	< 0.5^a,b,c^
Peroxide value (mequi/kg oil)	11.7	10.0	–

^a^EN14214 (European biodiesel standard).

^b^ASTM D6751 (United States).

^c^Thailand^51–53^.

The basic parameters directly related to oxidation products including an acid number and a peroxide value were measured. There were several parameters related to oxidation products, but the two were able to be determined with minimum effort by titration. The acid number remained unaffected whereas the peroxide value became lower, signifying that H-FAME exhibited a lower oxidation rate corresponding to the increase in oxidation stability.

### Preliminary design for large-scale H-FAME production

With 100 W of input power to the DBD power supply and with the production rate of 300 mL for 5 h, the energy efficiency in the present experiment was only 66.7 W/L–h. No power was required for FAME heating or catalyst preparation/removal/regeneration. There was no cost for initial catalyst procurement either. For a large-scale production using this novel green and non-thermal DBD plasma hydrogenation, one could employ a large set of electrodes to treat a large FAME surface with a powerful DBD power supply. He and H_2_ gases could also be completely recycled using a simple recirculating pump operating at slightly above ambient conditions, with only occasional replenishment of H_2_ gas to account for the consumed hydrogen by the C=C bonds. To continuously maintain the optimal gas concentration, a residual gas analyzer (RGA) through a gas sampling port could be installed for analysis of partial pressures of gases, assisting precise and real-time addition of H_2_ into the system. Thus, the only major expenditures to produce H-FAME using this novel green technique are electricity, hydrogen gas and FAME. Also, this technique is safe. Even though 80% hydrogen gas concentration was used and would, at first thought, be prone to explosion, in the closed system with no oxidizer present in the reaction chamber, e.g., O_2_ gas or oxygen atoms in FAME or even in the electrode, the hydrogen gas cannot explode even with the presence of the microfilament discharges. Operating at slightly above one atmosphere would ensure no atmospheric oxygen gas seeping into the system. The reaction chamber and the gas system must also be sealed properly to prevent gas leakage as the hydrogen gas is flammable. Its lower and upper explosive limits in the air at room temperature and atmospheric pressure are 4.3 and 76.5 mol%, respectively^54^. A similar safety infrastructure to that of conventional catalysis will ensure a safe operation of the DBD plasma hydrogenation system.

## Conclusions

The constructed DBD plasma reactor was utilized to effectively hydrogenate palm FAME. The most suitable condition was 100 W input power, 1 mm gas-filled gap size, 80% H_2_, room temperature and 5 h of reaction time for 300 mL FAME in the absence of a catalyst. This caused the reduction of C18:2 and C18:3 by 47.1 and 100%, respectively. The reduction of C=C bonds enhanced the oxidation stability from 12.8 to 20 h along with the change in the cloud point from 13.5 to 16 °C. The low-temperature plasma treatment did not create *trans-*fatty acid methyl ester normally formed in catalytic hydrogenation. The green DBD plasma hydrogenation provided superior performances to catalysis and was much simpler, for it required no dedicated reactor and materials to withstand high pressure and high temperature. Moreover, this technique can eliminate the problems or processes associated with a physical catalyst such as catalyst deactivation, pellet breakup, filtration and regeneration requiring high energy in the process. This novel and green technology has a promising potential to be applied for the hydrogenation of other high-value liquids, as well as for large-scale H-FAME production.

## Supplementary Information

Supplementary Information.
