# Supplementary material for: Low-temperature and atmospheric pressure plasma for palm biodiesel hydrogenation
Source: Sci Rep. 2021 Jul 9;11:14224. doi: 10.1038/s41598-021-92714-x (PMC8270954; doi:10.1038/s41598-021-92714-x)
Supplement: Supplementary file 1 — Supplementary Information. [file 41598_2021_92714_MOESM1_ESM.docx]

Supplementary Materials for

Low-Temperature and Atmospheric Pressure Plasma for Palm Biodiesel Hydrogenation

Grittima Kongprawes, Doonyapong Wongsawaeng*, Kanokwan Ngaosuwan,

Worapon Kiatkittipong, Suttichai Assabumrungrat

*Corresponding author. Doonyapong.W@Chula.ac.th. Department of Nuclear Engineering, Faculty of Engineering, Chulalongkorn University, 254 Phayathai Road, Pathumwan, Bangkok 10330, Thailand.

Supplementary Materials for this manuscript include the following:


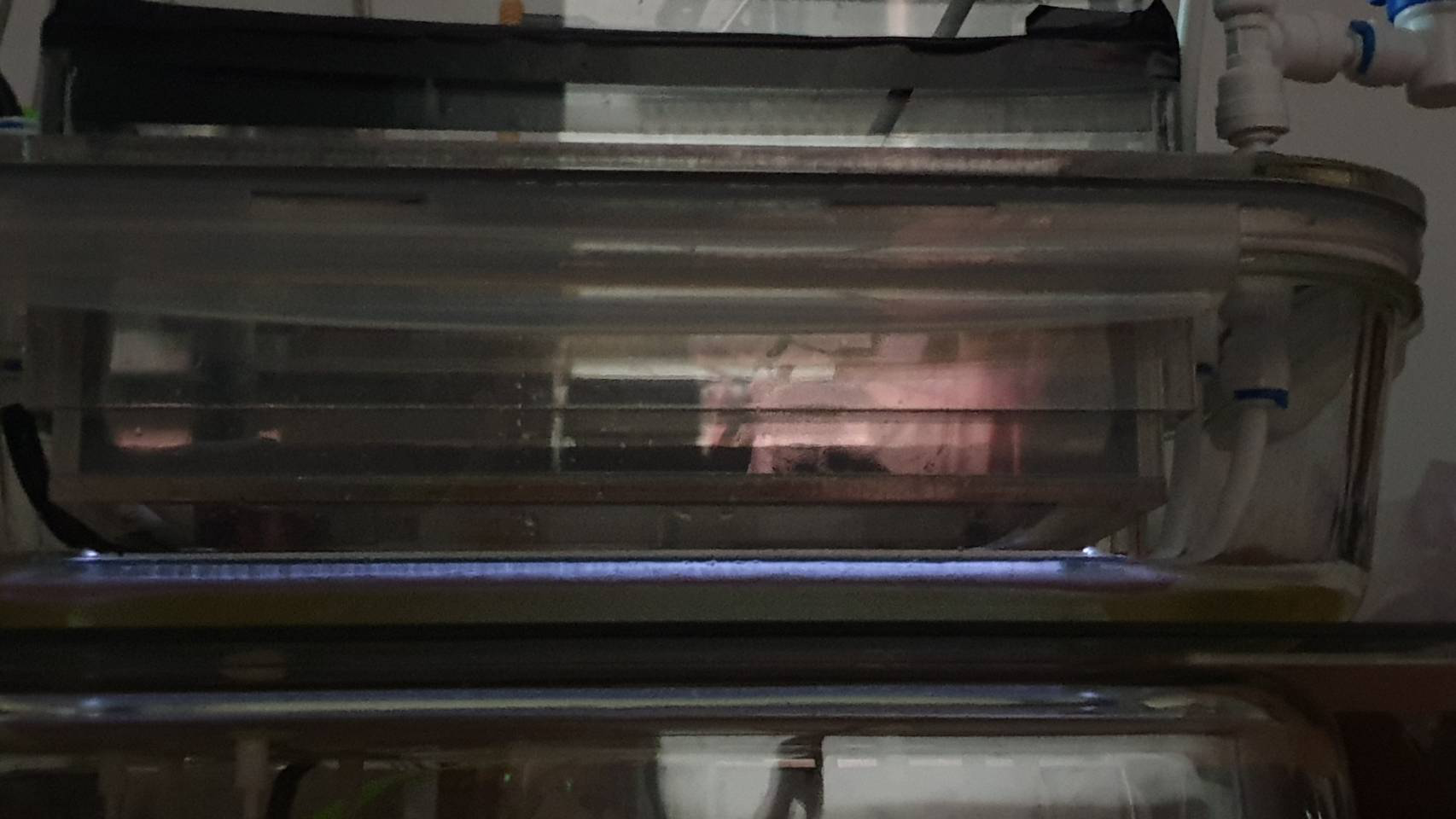


FAME inlet

Gas inlet

(a)


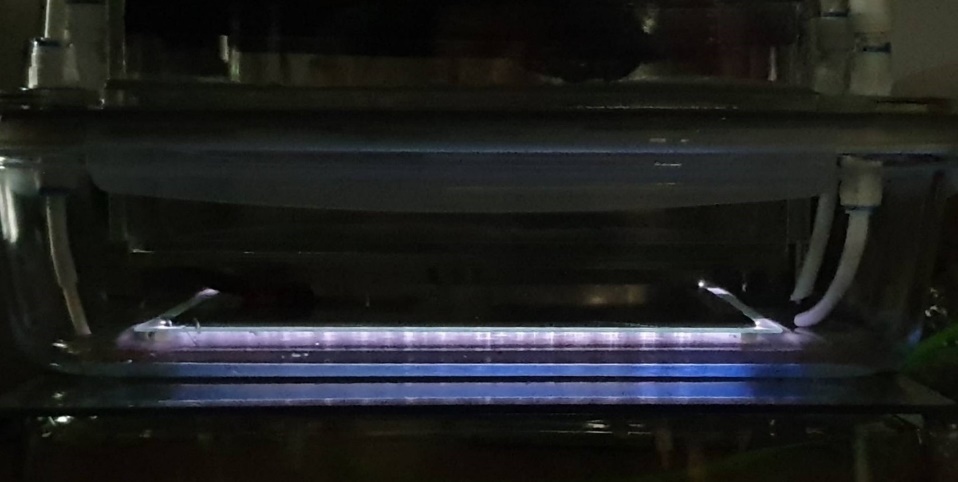


FAME inlet

FAME outlet

Gas inlet

(b)

**Fig. S1. Plasma generated (a) with FAME and (b) without FAME in chamber**

1. **Experimental investigation of plasma gas**

The constructed DBD plasma reactor could not generate plasma from pure H_2_, so an appropriate inert gas mixture was needed. He gas was chosen because plasma could be easily generated all over the electrode with few microfilament formations. N_2_ and Ar gases were also tested, but plasma could not be generated in the constructed reactor. We also tried to use N_2_ gas in a different DBD configuration without the glass dielectric attached to the upper electrode. In this configuration, the bottom of the glass container acted as a dielectric material. Plasma could be generated, but N_2_ plasma consisted of discrete large microfilament discharges at certain locations on the edges of the upper electrode compared to He gas, which was undesirable because of the very low plasma density and because microfilament discharges locally heated FAME in contact. With He gas, the diffuse plasma was observed all over the upper electrode, offering a much higher plasma density and much less intense microfilament discharges. As for using Ar gas, stable plasma could not be generated. Therefore, we decided to use the He/H_2_ mixed gas as can be seen in Fig. S2.

On an industrial scale, a simple gas recirculating system could be constructed and no He gas would be vented to the atmosphere. To maintain the optimal gas concentration, a residual gas analyzer (RGA) through a gas sampling port could be installed for analysis of partial pressures of gases, assisting precise and real-time addition of H_2_ into the system.


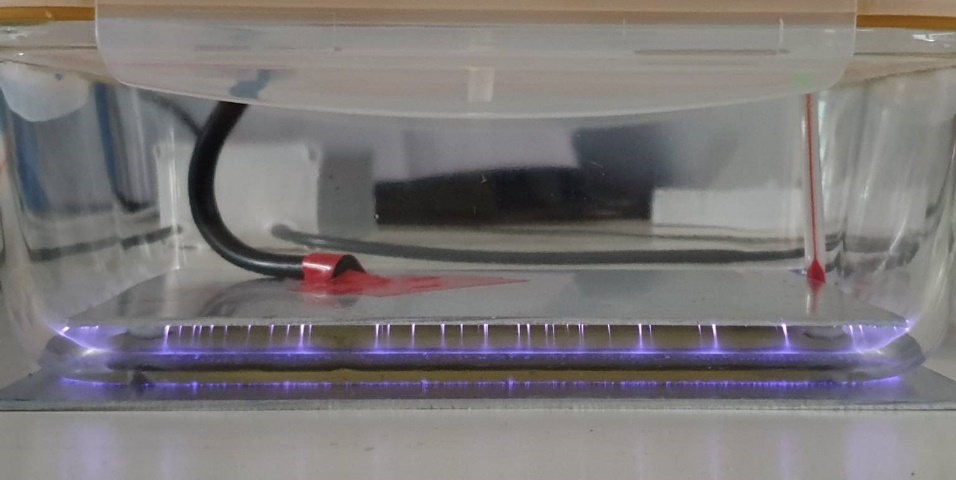


(a)


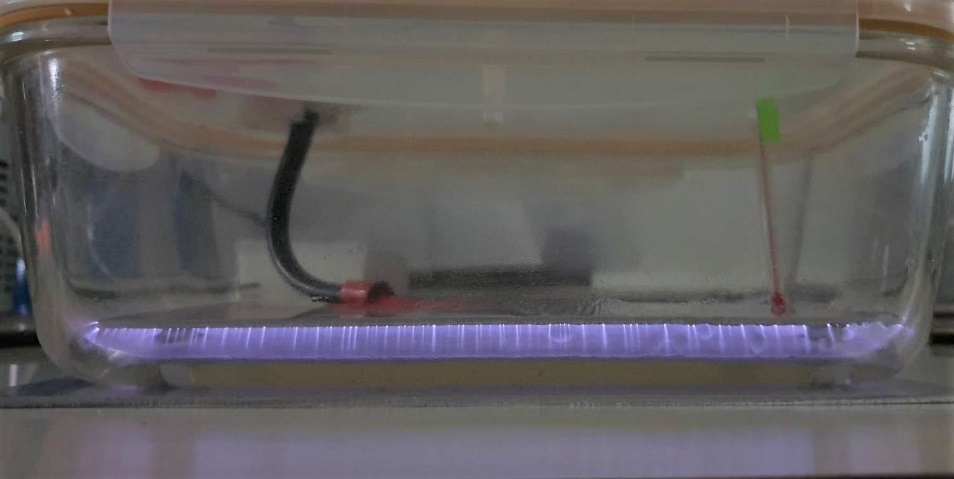


(b)

**Fig. S2. Generated plasma using different mixed gases (a) 25%H_2_ and 75% N_2_, (b) 25%H_2_ and 75%He (ambient temperature, 100 W input power)**

**
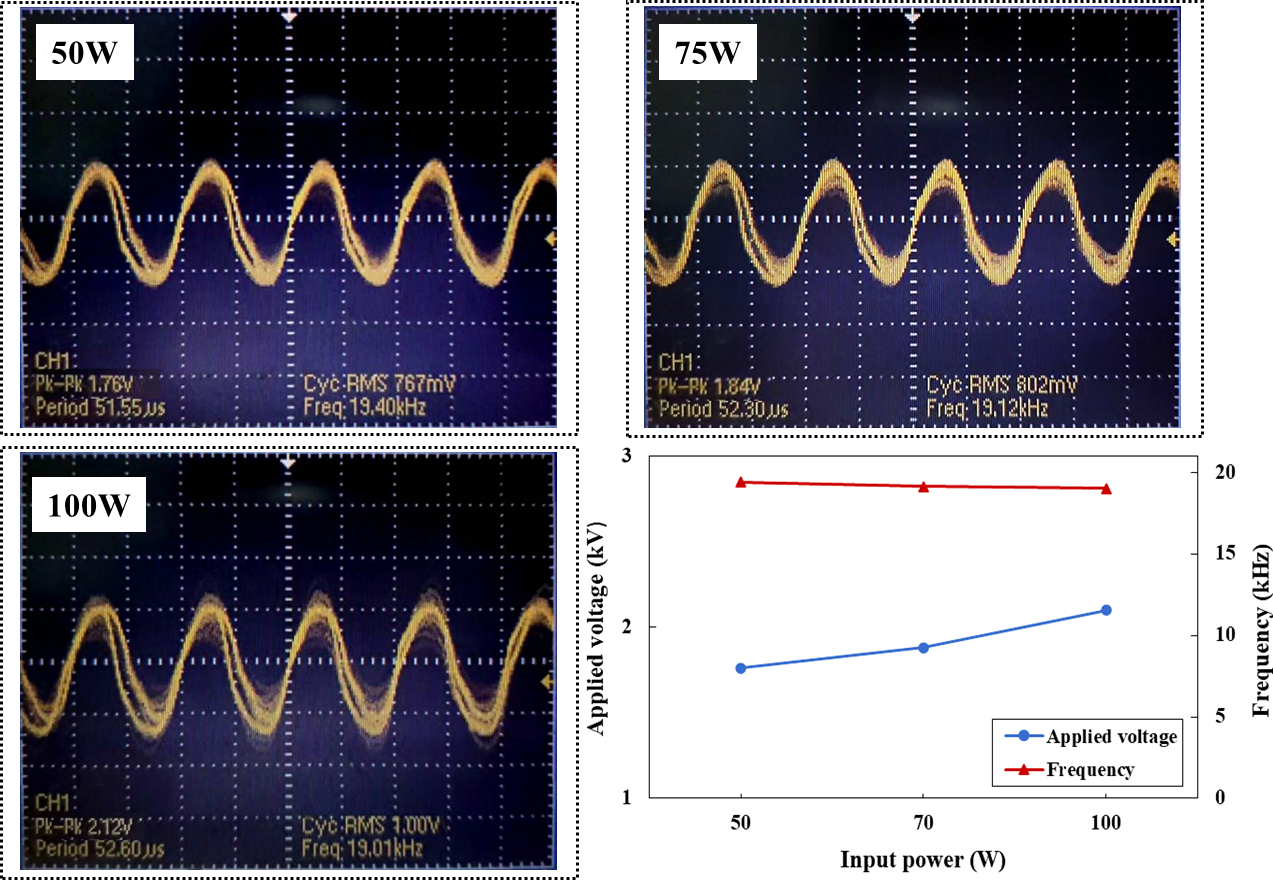
**

**Fig. S3. Discharge voltage characteristic of DBD plasma under different power levels (1 mm gap, 25%H_2_, room temperature)**

**Fig. S4. Discharge voltage under different gas-filled gap sizes**


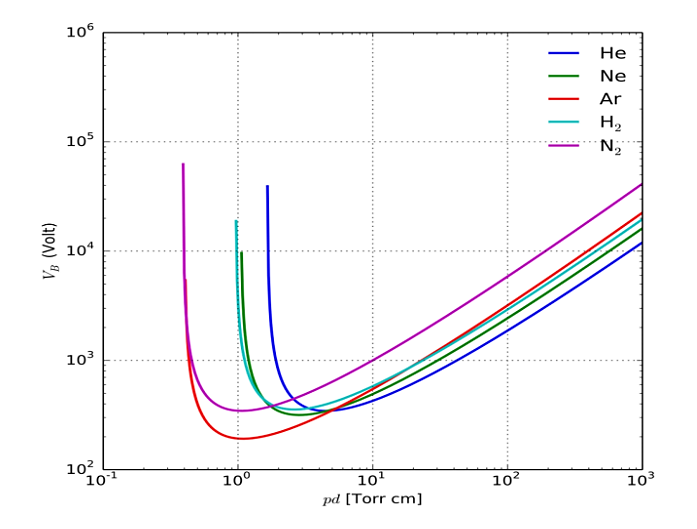


**Fig. S5.** **Paschen’s curve obtained for helium, neon, argon, hydrogen and nitrogen gases using expression for breakdown voltage as function of parameters p and d presented in Das et al.’s study^41^**


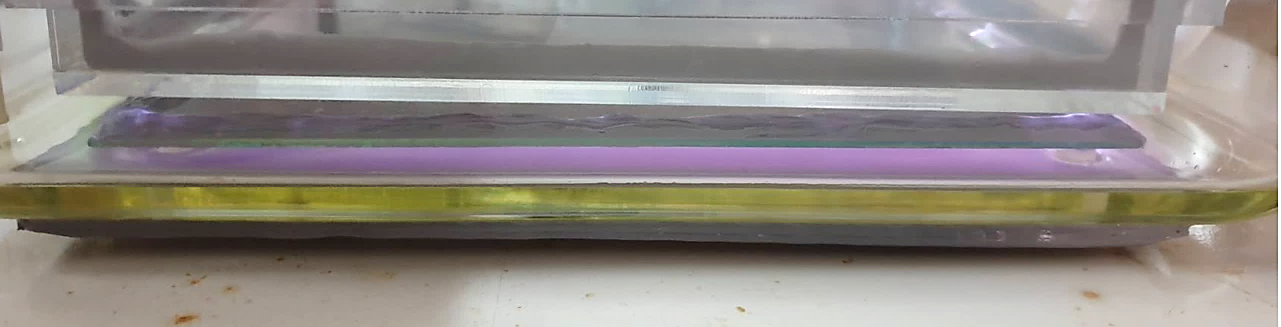


90%He + 10%H_2_


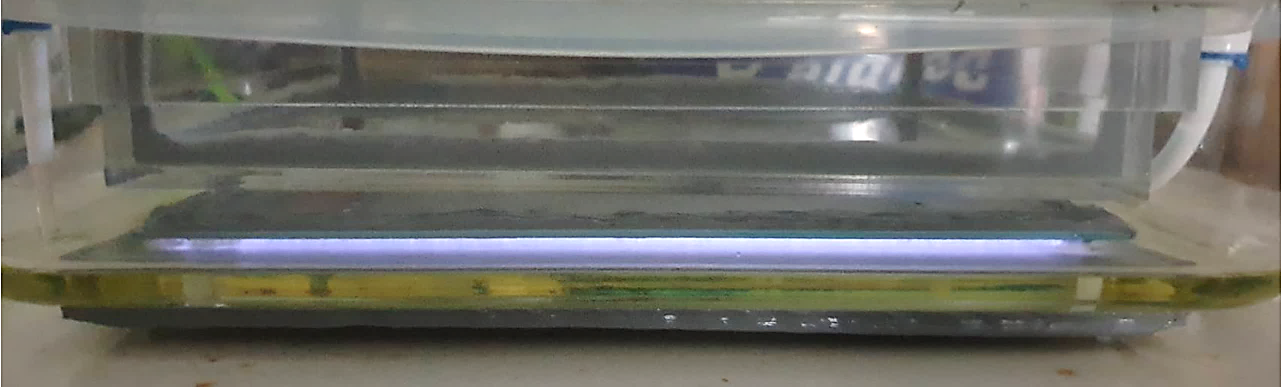


20%He + 80%H_2_

**Fig. S6. Plasma color under different mixed gas concentrations (100 W, 1 mm gap, room temperature)**
